# Identification of Textile Fibres Using a Near Infra-Red (NIR) Camera

**DOI:** 10.3390/jimaging11040096

**Published:** 2025-03-25

**Authors:** Fariborz Eghtedari, Leszek Pecyna, Rhys Evans, Alan Pestell, Stuart McLeod, Shan Dulanty

**Affiliations:** The Manufacturing Technology Centre, Ansty Business Park, Pilot Way, Coventry CV7 9JU, UK; leszek.pecyna@the-mtc.org (L.P.);

**Keywords:** NIR, spectroscopy, textiles, recycling, hyperspectral, carbon black dye, materials identification

## Abstract

Accurate detection of textile composition is a major challenge for textile reuse/recycling. This paper investigates the feasibility of identification of textile materials using a Near Infra-Red (NIR) camera. A transportable metric has been defined which could be capable of identification and distinction between cotton and polyester. The NIR camera provides a single data value in the form of the “intensity” of the exposed light at each pixel across its 2D pixel array. The feasibility of textile material identification was investigated using a combination of various statistical methods to evaluate the output images from the NIR camera when a bandpass filter was attached to the camera’s lens. A repeatable and stable metric was identified and was shown to be independent of both the camera’s exposure setting and the physical illumination spread over the textiles. The average value of the identified metric for the most suitable bandpass filter was found to be 0.68 for cotton, with a maximum deviation of 2%, and 1.0 for polyester, with a maximum deviation of 1%. It was further shown that carbon black dye, a known challenge in the industry, was easily detectable by the system, and, using the proposed technique in this paper, areas that are not covered by carbon black dye can be identified and analysed.

## 1. Introduction

Recycling of metals and plastics has been common practice for many years, as the benefits can be realised environmentally as well as commercially. Hyperspectral camera systems have been identified as a robust technology in the classification of plastic materials [1,2,3,4]. The fashion industry produces a huge number of textiles every year, and increasing the recycling of textiles will have a significant environmental impact. However, historically, the financial benefits have not been significant enough to make this commercially viable. With the introduction of new environmental legislation, recycling is becoming economically more viable. To add more value to the recycled textiles, they must be separated into batches of known materials. A large proportion of the labels on the textiles cannot be relied upon to provide the correct information about the materials’ composition [5], so the labels cannot be trusted for recycling.

Automated sorting systems employ vision systems for material identification. These systems include single NIR (Near Infra-Red) sensors, where each sensor inspects a small area of the textile, as well as hyperspectral systems where the imaging system scans a line across the moving textiles, normally placed over a moving conveyor system.

Hyperspectral systems are normally used for identification of materials in multiple recycling streams. The hyperspectral system provides higher number of data wavelength points (typically 224) over the range of the light frequency it covers. This means high number of data values for every pixel in the image, creating a characteristics signature for what has been sampled. Another technology that could be employed in sorting materials in multiple recycling streams is NIR cameras; while NIR single spectroscopy sensors provide a spectrum response of light wavelengths in a small finite region, NIR cameras provide a 2D grayscale image of the area within the camera’s field of view. The cost of an NIR camera system is generally one-fifth of a hyperspectral system. The main issue in the adoption of NIR cameras for textile identification is that very limited data are captured at each camera pixel, which is in the form of light “intensity”, also referred to as brightness. The images acquired by the NIR camera provide the reflected light intensities from an object in its field of view; therefore, the system is very sensitive to the intensity of the incident light and the consequent reflected lights. For widespread adoption of NIR cameras in “real world” environmental settings, algorithms and procedures are required to provide metrics which allow the use of the system independent of the camera’s exposure settings and the system’s physical lighting setup.

This paper investigates the feasibility of using an NIR camera as an alternative, cost-effective technology for identification of textile materials. A solution for minimising the effects of non-uniform illumination across the camera’s field of view is proposed with no dependencies on the camera exposure settings or the light spread across the image. This methodology and the proposed use of a robust and repeatable metric that can be used to identify the constituent material of the textile samples under inspection are the novel contributions to knowledge in this paper.

The NIR camera used in this investigation is an area sensor with a global shutter and a resolution of 1280 by 1024 pixels. Its field of view is determined by the camera lens, and this parameter can be easily changed according to the operational requirements, and, unlike the hyperspectral systems, it does not require any relative displacements of the object under examination with respect to the camera.

The work in this project was a part of the Automatic-Sorting for Circular Textiles (ACT) consortium, funded by Innovate UK (IUK) research. The consortium aims to support and promote circularity in the UK’s textiles industry.

## 2. Related Works

### 2.1. NIR Spectroscopy for Textile Classification

Most research conducted on material identification utilises spectroscopy, where a few (multispectral) or high number (hyperspectral) of bands are used. It is uncommon to use non-spectral 2D NIR cameras with a bandpass filter for material identification—as is presented in this paper. As mentioned in the introduction, the spectroscopy methods are commonly used for identification of plastic materials [1,2,3,4]. These methods have also emerged as promising alternatives for textile classification due to their non-destructive nature, rapid analysis capabilities, and potential for cost-effectiveness. Particularly, NIR spectroscopy operates in the wavelength range of 780 nm to 1700 nm, which is effective in identifying specific chemical groups existing in fibres due to their NIR reflective properties (e.g., C-H, O-H, C-O in cellulose) [6]. This makes NIR spectroscopy suitable for differentiating various textile fibres, including natural fibres, like cotton, wool, or silk, and synthetic like polyester and nylon. Several studies have shown the effectiveness of NIR hyperspectroscopy in textile materials identification [6,7,8]. Another spectral range often used and mentioned in the literature for fibre identification is Short-Wave Infrared (SWIR)) [6,9], which often operates in the range of 1000 nm to 2500 nm [9]. The range limits for the NIR and SWIR are not strictly defined, and different ranges are quoted by different sources [10,11].

The hyperspectral data are often processed by a variety of Machine Learning methods [12] including deep-learning algorithms such as Convolutional Neural Networks that perform the classification [8]. These algorithms allow the extraction of characteristic features from the spectrum of specific fibres. These methods operate on hyperspectral data which require expensive cameras or scanners. There were some attempts to reduce the cost by choosing an NIR multispectral sensor (with lower spectral resolution compared to hyperspectral sensors), e.g., a Hamamatsu C15713 which operates in the range 1500–1859 nm with a spectral resolution of 20 nm [13].

The method presented within this paper is based on use of relatively low cost and an easily deployable NIR camera producing only a single intensity value per pixel. Unlike the methods used in spectroscopy-based systems, here, statistical-based methods are proposed which are robust and easier to implement without the need for any training or development of machine learning-based models.

### 2.2. Addressing Non-Uniform Illumination

One of the challenges in using NIR cameras for textiles differentiation is non-uniform illumination across the camera’s field of view. This can lead to inconsistent spectral data. There have been many approaches proposed to mitigate the non-uniform illumination problem. These methods vary from the utilisation of an L2-Norm based on Retinex theory [14] to the implementation of a specific neural network that is trained to normalise the images [15,16]. Most methods focus on the processing of standard images (e.g., with humans or animals) and are often based on colour image processing (such as [14]), hence cannot be directly applied to the problem presented. The neural network methods [15,16] are relatively resource- and time-costly, as they require training the machine learning model. The normalisation method proposed in this paper is relatively straightforward to implement and apply to textile NIR images.

## 3. Methods

### 3.1. Removal of the Effects of Non-Uniform Illumination

**Figure 1:**
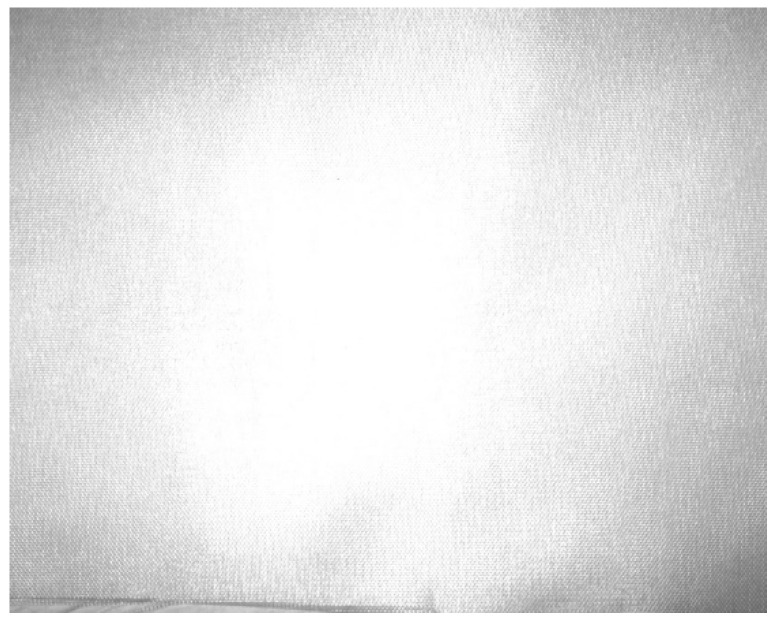
Image with saturated pixels.

The light source for the NIR camera must cover the camera’s sensitivity range, which is from 400 nm to 1700 nm. In this investigation, two halogen lights were used to provide illumination. Figure 1 shows an extreme case of uneven, non-homogeneous illumination across the camera’s field of view. Creating absolute uniform lighting conditions and providing high intensity illumination is not typically feasible. In most cases, the illumination is not uniform, which is also true in our experimental setup. This is very challenging, as the output from the camera is primarily dependent on light intensity. The camera outputs grayscale images, where the intensity of the pixel is a function of the spectral reflectance of the material under observation and the incident light. This paper proposes a method to increase the usability of NIR cameras in the current application by performing corrective actions on images captured by the NIR camera to greatly reduce or remove the effects of non-homogenous illumination.

#### 3.1.1. Calculating a Measure of Uneven Illumination

In our experimental setup, it is not possible to create a uniform illumination using two halogen lamps. Figure 1 shows an example of the illumination in an extreme case, where parts of the image are overexposed, when capturing images of an optical white tile. This image will be treated as the reference image for subsequent images captured under the same lighting conditions, camera, and lens settings.

The aperture and exposure time must be set such that there are no, or the least number of, overexposed and underexposed pixels in the image, as much as possible. In this case, a white overexposed pixel value would be 255. Extreme underexposed pixels with a value of 0 are also undesirable and, similarly to the extremely overexposed pixels, result in the reduction of the number of usable pixels in the subsequent images taken under the same conditions.

**Figure 2:**
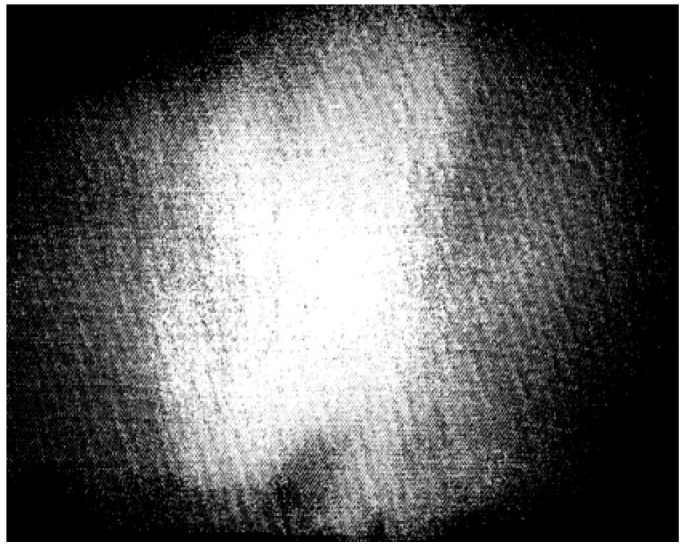
Saturated pixels highlighted.

To highlight and warn the operator of the existence of overexposed (value of 255) and underexposed (value of 0) pixels when capturing reference images, post-processing software has been developed to highlight these pixels. Figure 2 shows the same image from Figure 1, with the post-processing software applied, highlighting many overexposed pixels.

The illumination, or, in this case, the camera and lens settings, must be adjusted to minimize or remove any of the undesirable pixels. In an ideal situation, the processed reference image should be completely black.

Once an acceptable reference image is captured, the mean intensity value of the pixels in the image is calculated. In this calculation, the overexposed and underexposed pixels are excluded. If the pixel value = 0 or =255, then it is excluded from the calculations below:

(1) $$m_{r}=\frac{\sum V_{r}\left(i,j\right)}{n_{r}}$$

where

i: image row;
j: image column;
$V_{r}\left(i,j\right)$: pixel value at row i, column j, in the reference image;
$n_{r}$: total number of non-excluded pixels in the reference image;
$m_{r}$: average value of non-excluded pixels in the reference image.

Using the average pixel value of the non-excluded pixels in the reference image, an array is populated with the values of the differences in the actual value of the pixel from the calculated mean value of the non-excluded pixels population:

(2) $$V_{∆}\left(i,j\right)=m_{r}-V_{r}\left(i,j\right)$$

where

$V_{∆}\left(i,j\right)$: difference value for the pixel at row i column j from the average value of non-excluded pixels in the reference image.

#### 3.1.2. Image Correction Due to Uneven Illumination

Equation (2) can be used on the subsequent images captured under the same illumination settings as that of the reference image, to reduce the effects of the non-uniform illumination on the captured images. Using the process below, the value of the pixels is adjusted to compensate for the non-uniformity in the illumination.

For all non-excluded pixels

(3) $$V_{c}\left(i,j\right)=V_{n}\left(i,j\right)+V_{∆}\left(i,j\right)$$

$if\;V_{c}\left(i,j\right)\leq 0\;\to V_{c}\left(i,j\right)=0$

$if\;V_{c}\left(i,j\right)\geq 255\;\to V_{c}\left(i,j\right)=0$

where

$V_{n}\left(i,j\right)$: pixel value at row i, column j, in the new image;
$V_{c}\left(i,j\right)$: corrected, pixel value at row i, column j.

**Figure 3:**
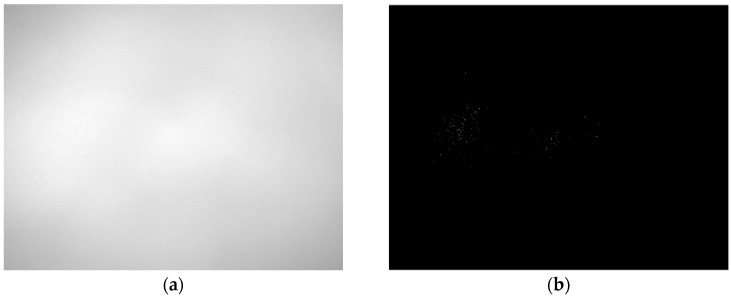

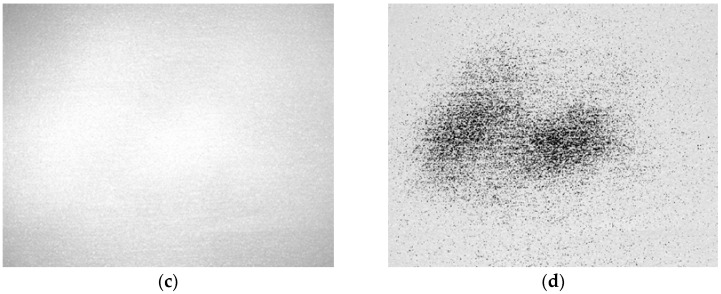
Exclusion of overexposed pixels in the corrected image: (**a**) reference image; (**b**) reference image’s overexposed pixels; (**c**) overexposed target image; (**d**) corrected image’s overexposed pixels.

In Figure 3, the overexposed pixels that were present in the reference image, as well as any overexposed pixels in the target image, are set to a value of 0 and will be excluded from any further processing. The same would also be true for any extreme underexposed pixels with a value of 0.

### 3.2. Processing Images of Textile Samples

The aim of this study is to process the images of textile samples captured by the NIR camera and extract the values of parameters which could be used to determine the constituent material of the textile samples. In order to define the metrics for the identification of the textile materials using the NIR camera, we require the following requirements to be met:

- The process must be able to cater for non-homogeneity of the illumination.
- Repeatable and transportable metrics must be identified that would not be affected by the camera exposure time or the physical light spread.

As described above, once the setup of the halogen lamps, camera, and lens settings, have been adjusted and set, a one-off reference image for that configuration is captured. The pixels in the reference image are processed to calculate the parameters and arrays as described by Equations (1)–(3).

A textile test sample is placed in the camera’s field of view replacing the optical white tile, and an image is captured. Following uneven illumination adjustments, the captured image is processed to calculate several parameters which may be used as indicators for the determination of the material of the textile sample being inspected.

(4) $$\mu_{c}=\frac{\sum V_{c}\left(i,j\right)}{N}$$

where

$\mu_{c}$: population mean of the pixels in the corrected image;
N: size of the population of the corrected image.

If $V_{c}\left(i,j\right)$ = 0 or =255, it is excluded from the calculations.

Next, the standard deviation of pixel values in the corrected image is calculated.

(5) $$\sigma_{c}=\sqrt{\frac{\Sigma {\left(V_{c}\left(i,j\right)-\mu_{c}\right)}^{2}}{N}}$$

where

$\sigma_{c}$: standard deviation of the corrected image.

If the pixel value = 0 or =255, it is excluded from the calculations.

Furthermore, the pixel values in the corrected image and the reference image are normalised. Here, we have used two types of normalisations, Min-Max normalisation and Z-Score normalisation.

(6) $$V_{min\_max}\left(i,j\right)=\frac{V_{c}\;\left(i,j\right)-V_{c\_min}}{V_{c\_max}-V_{c\_min}}$$

(7) $$\mu_{min\_max}\left(i,j\right)=\frac{\sum V_{min\_max}\left(i,j\right)}{N}$$

(8) $$\sigma_{min\_max}=\sqrt{\frac{\Sigma {\left(V_{min\_max}\left(i,j\right)-\mu_{min\_max}\right)}^{2}}{N}}$$

where

$V_{min\_max}\left(i,j\right)$: Min_Max corrected normalised pixel value at row i, column j;
$\mu_{min\text{\_}max}$: mean pixel value for the Min_Max normalised corrected image;
$\sigma_{min\text{\_}max}$: standard deviation of the Min_Max normalised corrected image.

If the pixel value = 0 or =255, it is excluded from the calculations.

(9) $$V_{Z\text{\_}Score}\left(i,j\right)=\frac{V_{c}\left(i,j\right)-\mu_{c}}{\sigma_{c}}$$

(10) $$\mu_{Z\text{\_}Score}=\frac{V_{Z\text{\_}Score}\left(i,j\right)}{N}$$

(11) $$\sigma_{Z\text{\_}Score}=\sqrt{\frac{\sum {\left(V_{Z\text{\_}Score}\left(i,j\right)\right)}^{2}}{N}}$$

where

$V_{Z\text{\_}Score}\left(i,j\right)$: Z_Score normalised corrected image, pixel value at row i, column j;$\mu_{Z\text{\_}Score}$: mean pixel value for the Z_Score normalised corrected image;
$\sigma_{Z\text{\_}Score}$: standard deviation of the Z_Score normalised image.

Other parameters considered are as follows:

(12) $$m_{Min\text{\_}Max}=\frac{\sum V_{r\text{\_}Min\text{\_}Max}\left(i,j\right)}{n_{r}}$$

(13) $$m_{Z\text{\_}Score}=\frac{\sum V_{r\text{\_}Z\text{\_}Score}\left(i,j\right)}{n_{r}}$$

where

$m_{Min\text{\_}Max}$: mean of the Min_Max normalised reference image;
$m_{Z\text{\_}Score}$: mean of the Z_Score normalised reference image;
$V_{r\text{\_}Min\text{\_}Max}\left(i,j\right)$: pixel value of the Min_Max normalised reference image, at row i, column j;
$V_{r\text{\_}Z\text{\_}Score}\left(i,j\right)$: pixel value of the Z_Score normalised reference image, at row i, column j.

(14) $$\sigma_{r}=\sqrt{\frac{\sum {\left(V_{r}\left(i,j\right)-m_{r}\right)}^{2}}{n_{r}}}$$

(15) $$\sigma_{Min\text{\_}Max}=\sqrt{\frac{\sum {\left(V_{r\text{\_}Min\text{\_}Max}\left(i,j\right)-m_{r\text{\_}Min\text{\_}Max}\right)}^{2}}{n_{r}}}$$

(16) $$\sigma_{r\text{\_}Z\text{\_}Score}=\sqrt{\frac{\sum {\left(V_{r\text{\_}Z\text{\_}Score}\left(i,j\right)-m_{r\text{\_}Z\text{\_}Score}\right)}^{2}}{n_{r}}}$$

where

$\sigma_{r}$: standard deviation of the reference image;
$\sigma_{Min\text{\_}Max}$: standard deviation of the Min_Max normalised reference image;
$\sigma_{r\text{\_}Z\text{\_}Score}$: standard deviation of the Z_Score normalised reference image.

(17) $$M_{r\text{\_}c}=\frac{\mu_{c}}{m_{r}}$$

(18) $$M_{r\text{\_}Min\text{\_}Max}=\frac{\mu_{min\text{\_}max}}{m_{r}}$$

(19) $$M_{r\text{\_}Z\text{\_}Score}=\frac{\mu_{Z\text{\_}Score}}{m_{r}}$$

where

$M_{r\text{\_}c}$: relative mean value of the corrected image to the mean of the reference image;
$M_{r\text{\_}Min\text{\_}Max}$: relative mean value of the Min_Max image to the reference Min_Max image;
$M_{r\text{\_}Z\text{\_}Score}$: relative mean value of the Z_Score image to the reference Z_Score reference image.

(20) $$\sigma_{R}=\frac{\sigma_{c}}{m_{r}}$$

(21) $$\sigma_{R\text{\_}Z\text{\_}Score}=\frac{\sigma_{Z\text{\_}Score}}{m_{r}}$$

(22) $$\sigma_{R\text{\_}Min\text{\_}Max}=\frac{\sigma_{Min\text{\_}Max}}{m_{r}}$$

(23) $$\sigma_{R\text{\_}ref\text{\_}STD}=\frac{\sigma_{r}}{m_{r}}$$

where

$\sigma_{R}$: relative standard deviation of the corrected image to reference image mean value;
$\sigma_{R\text{\_}Min\text{\_}Max}$: relative standard deviation of the Min_Max image to reference image mean value;
$\sigma_{R\text{\_}Z\text{\_}Score}$: relative standard deviation of the Z_Score image to reference image mean value;
$\sigma_{R\text{\_}ref\text{\_}STD}$: relative standard deviation of the corrected image to reference image mean value.

(24) $$\sigma_{R\text{\_}ref\text{\_}STD\text{\_}Min\text{\_}Max}=\frac{\sigma_{Min\text{\_}Max}}{\sigma_{r}}$$

(25) $$\sigma_{R\text{\_}ref\text{\_}STD\text{\_}Z\text{\_}Score}=\frac{\sigma_{Z\text{\_}Score}}{\sigma_{r}}$$

(26) $$\sigma_{R\text{\_}STD\text{\_}Min\text{\_}Max}=\frac{\sigma_{Min\text{\_}Max}}{\sigma_{c}}$$

(27) $$\sigma_{R\text{\_}STD\text{\_}Z\text{\_}Score}=\frac{\sigma_{Z\text{\_}Score}}{\sigma_{c}}$$

where

$\sigma_{R\text{\_}ref\text{\_}STD\text{\_}Min\text{\_}Max}$: relative standard deviation of the Min_Max image to standard deviation of the reference image;
$\sigma_{R\text{\_}ref\text{\_}STD\text{\_}Z\text{\_}Score}$: relative standard deviation of the Z_Score image to standard deviation of the reference image;
$\sigma_{R\text{\_}STD\text{\_}Min\text{\_}Max}$: relative standard deviation of the Min_Max image to standard deviation of the corrected image;
$\sigma_{R\text{\_}STD\text{\_}Z\text{\_}Score}$: relative standard deviation of the Z_Score image to standard deviation of the corrected image.

### 3.3. Experimental Process for Determination of Suitable Metrics for Textile Materials Identification

Images were captured at 10 different camera exposure times, for each of the three bandpass filters and each of the four textile samples, totalling 120 images. For each filter and camera exposure time, an image of an optical white plate was captured and used as the reference image.

The exposure times used resulted in a wide range of image brightnesses. The exposure time range settings are different for the other two filters, as it is dependent on the amount of light that each filter allows to pass through at specific light wavelengths. The exposure time ranges set for the other filters similarly cover a broad range of image brightnesses as above.

The image capture procedure followed for each bandpass filter is as follows:

- Set the exposure time for the specific bandpass filter.
- Capture a reference image.
- Capture images of the four textile samples, two 100% cotton samples and two 100% polyester samples.
- Repeat the process above over 10 exposure time settings.
- The exposure settings ranges used for the three filters were as follows:
- for Bi1300 nm: 4000 to 44,000 µs;
- for Bi1550 nm: 7000 to 40,000 µs;
- for BP1550 nm:1000 to 10,000 µs.

Using the Equations (1)–(3), the reference image was used to correct the illumination spread of the captured sample images. Using the Equations (1), (4)–(27), the metrics described by those equations were calculated.

## 4. Results

### 4.1. Experimental Setup

**Figure 4:**
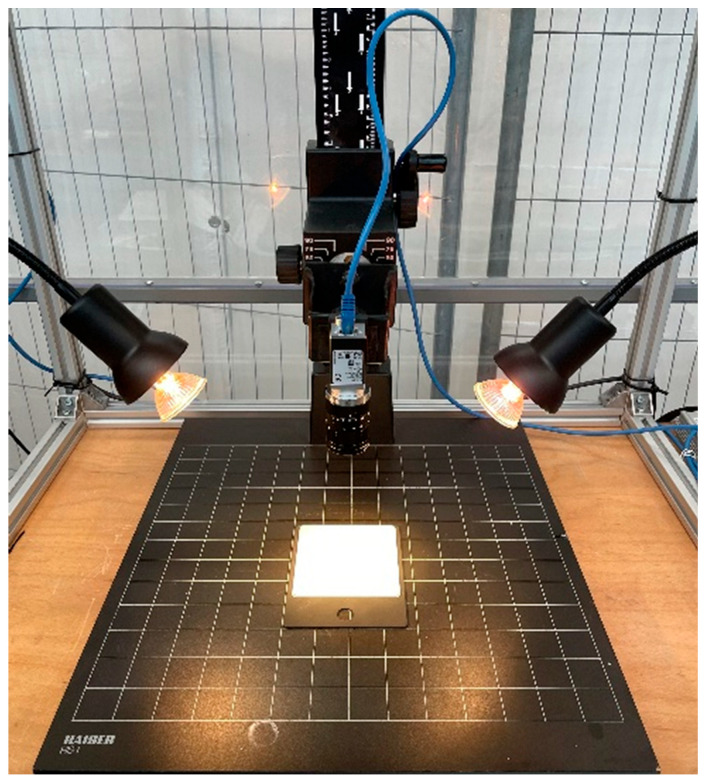
Experimental setup.

Figure 4 shows the hardware setup used for capturing images. The camera used is a Basler a2A1280-80gmSWIR [17]. It houses a Sony IMX990 sensor with a resolution of 1.3 MP (1280 px by 1024 px) and a maximum frame rate of 80 frames per second. The sensor is sensitive to the wavelength range of 400 nm to 1700 nm. The lens used with this camera is a 12 mm Computar M1218-APVSW [18]. Two 35-Watts halogen lamps were used for illumination of the test pieces.

**Figure 5:**
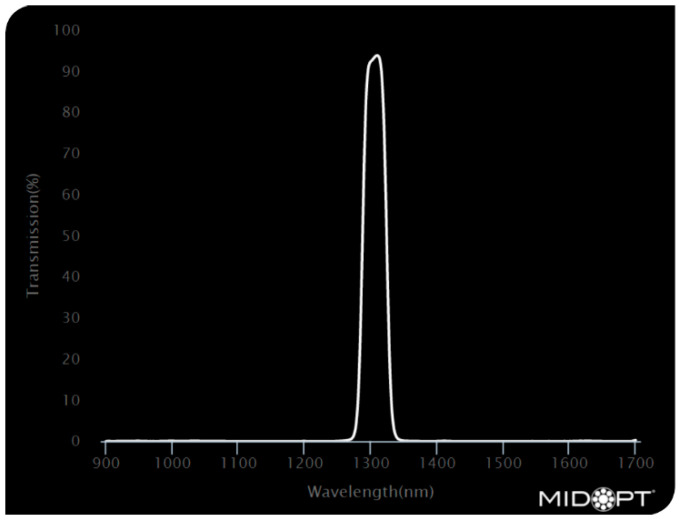
Bi1300—Short-Wave Infrared Bandpass filter [19].

**Figure 6:**
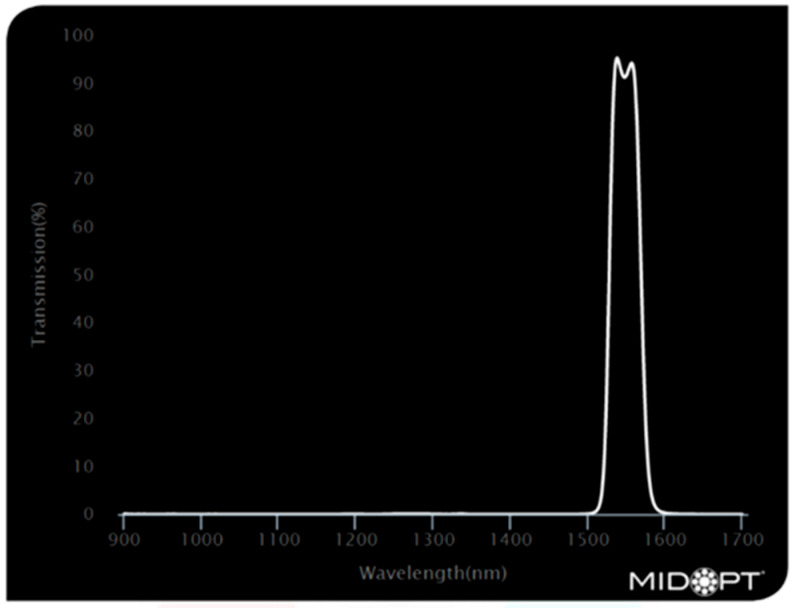
Bi1550—Short-Wave Infrared Bandpass filter [20].

**Figure 7:**
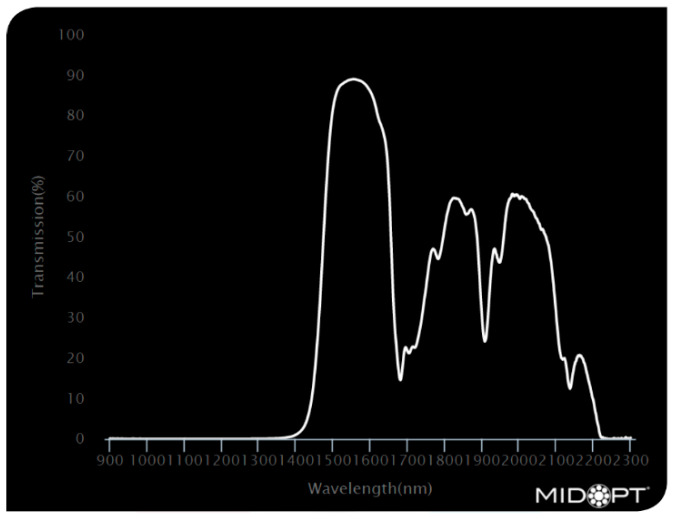
BP1550—Short-Wave Infrared Bandpass filter [21].

Three bandpass filters were used to investigate the variations in the camera’s response when looking at textile samples. The filters used were Bi1300 [19], Bi1500 [20], and BP1500 [21]. Figure 5, Figure 6 and Figure 7 show the filters optical characteristics.

**Figure 8:**
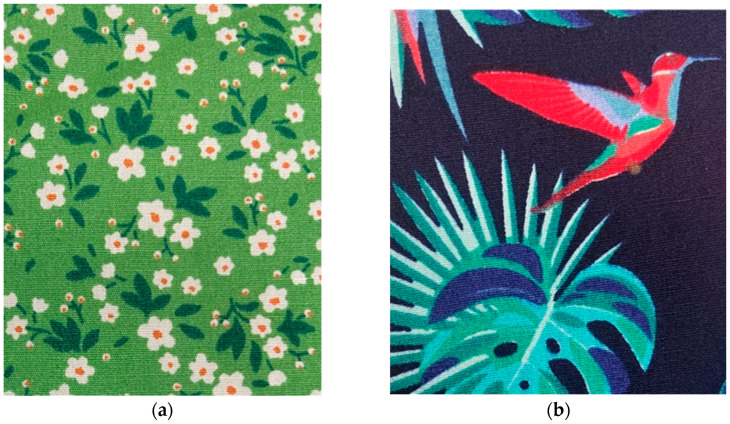

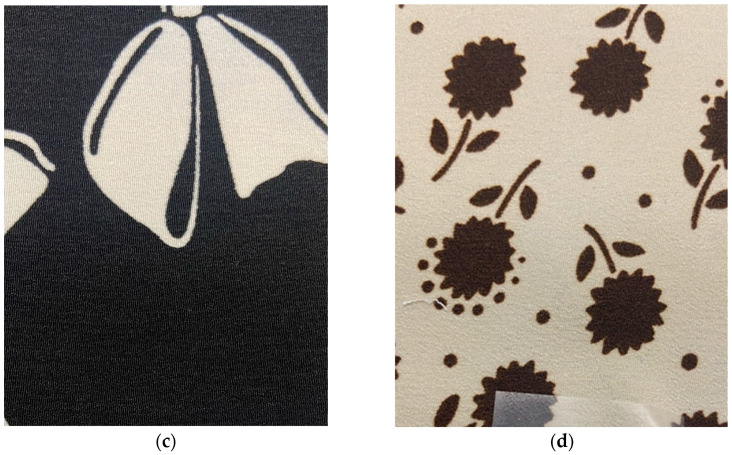
The four textile samples that were used for the investigations: (**a**) AA23—100% cotton; (**b**) AA24—100% cotton; (**c**) AA34—100% polyester; (**d**) AA36—100% polyester.

Four textile samples, two 100% cotton and two 100% polyester, were used to conduct the investigation. Figure 8 shows the four textile samples that were used for the investigations.

**Figure 9:**
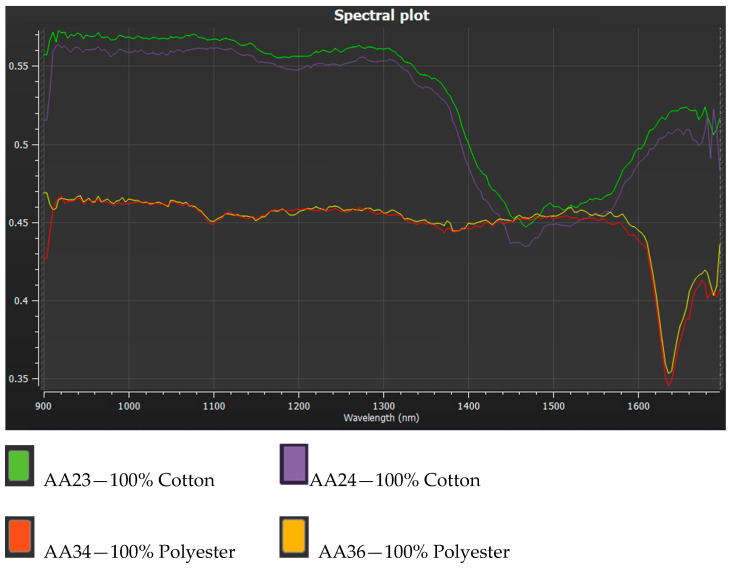
The hyperspectral response for the four textile samples.

Figure 9 shows the spectrum responses obtained from a hyperspectral system for the four textile samples. The cotton samples have similar spectral characteristics as do the polyester samples. The dips in the spectral characteristics can be used to help to partially identify the material under inspection by using appropriate bandpass filters.

### 4.2. Experimental Results

#### 4.2.1. Effects of the Exposure Time

**Figure 10:**
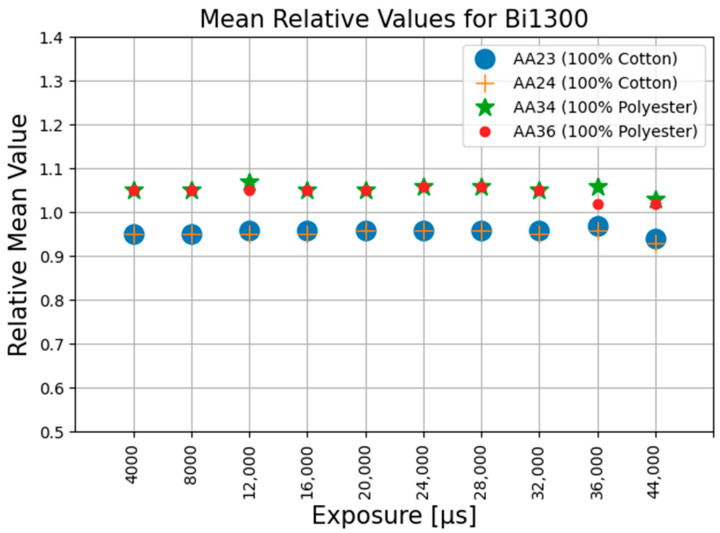
Mean relative values using Bi1300 filter at different exposures.

**Figure 11:**
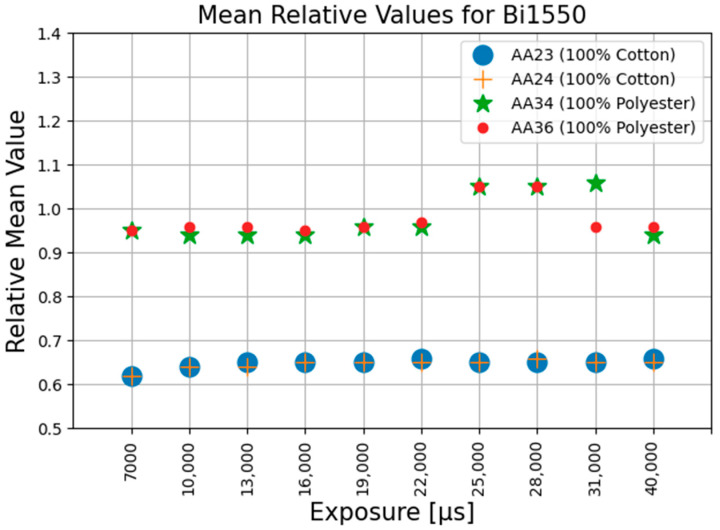
Mean relative values using Bi1550 filter at different exposures.

**Figure 12:**
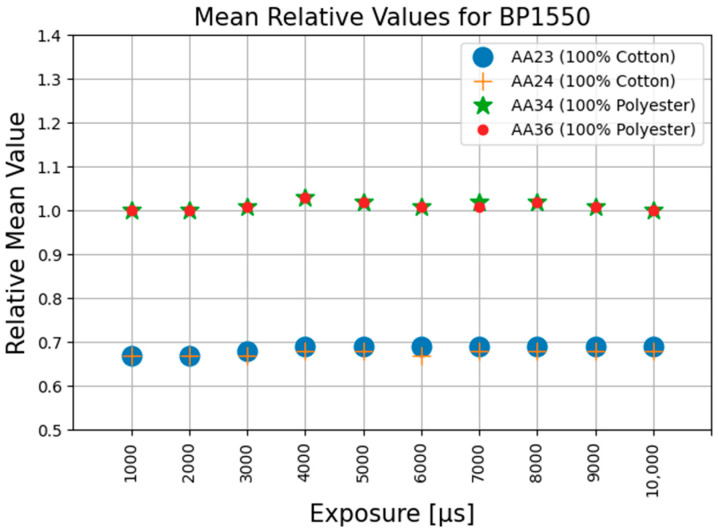
Mean relative values using BP1550 filter at different exposures.

The values for all the parameters as described in the Equations (1)–(27) were all calculated and were tabulated for further analysis. It was observed that the $M_{r\text{\_}c},$ the ratio of the mean pixel value of corrected image to the mean pixel value of the reference image, from Equation (17), remained almost constant for the same materials and bandpass filter over the range of the exposure times used. No other repeatable patterns could be observed within the other calculated parameters. Figure 10, Figure 11 and Figure 12 graphically demonstrate the results of the $M_{r\text{\_}c}$ for the three filters at different exposure time settings.

The average value of $M_{r\text{\_}c}$ for 100% cotton is 0.68 and 0.65 for the BP1550 and Bi1550 filters, respectively, with maximum deviations of 2% and 4%. The average value of $M_{r\text{\_}c}$ for 100% polyester is 1.0 and 0.98 for the BP1550 and Bi1550 filters, respectively, with maximum deviations of 1% and 8%. For both filters, the separation in the mean value of the $M_{r\text{\_}c}$ for the fibre type means that the value of $M_{r\text{\_}c}$ can be used as a valid metric, distinguishable from noise, for identification of these fibre materials. The results are presented in Table 1.

In the case where the Bi1300 filter was used, the value of $M_{r\text{\_}c}$ remains reasonably constant; however, the separation between the values of this parameter for 100% cotton and 100% polyester is very small; thus, any noise can significantly affect the outcome of the identification when using the Bi1330 bandpass filter.

The experimental results show that the value of $M_{r\text{\_}c}$, the ratio of the mean value of the corrected image to the mean of the reference image, from Equation (17), was not affected by the changes in the camera exposure time.

#### 4.2.2. Effects of Light Source Physical Positioning

**Figure 13:**
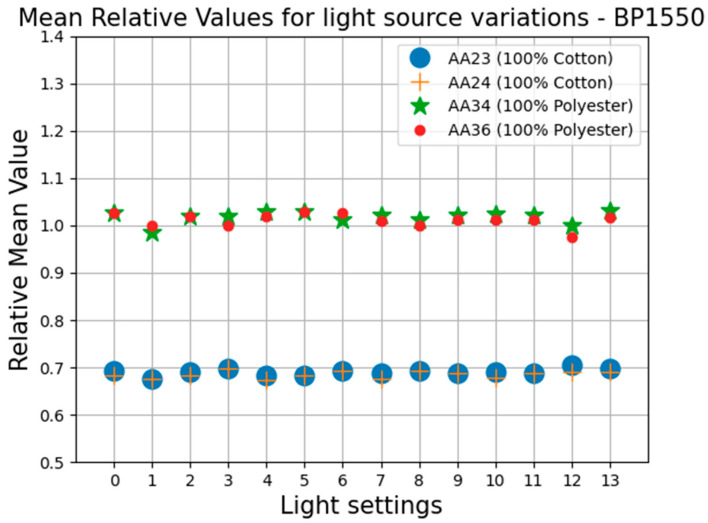
Relative mean values when the light sources positions are changed.

In this section, tests are carried out to see if the lack of dependency of this parameter to the camera’s exposure settings will also hold true when physically moving and adjusting the light sources while keeping the exposure time unchanged. For this investigation, only one filter, the BP1550 bandpass filter, was used, as, if this relationship holds for one filter, it follows the previous results that it would also hold for the other filters being tested. This is because the light’s physical positioning would similarly affect the pixels’ intensity values. Reference images were captured of an optical white surface at several arbitrary light source physical positions while keeping the exposure time unchanged between the changes. By physically adjusting the position and angles of the two light sources, the spread of the light changed across the images. Figure 13 graphically demonstrates the results when the two light sources used in the experimental setup were moved to arbitrary positions. The same four textile samples as in the previous tests were used.

As can be seen from the results for the value of $M_{r\text{\_}c}$ for the cotton and polyester test samples, calculated at various illumination settings using the BP1550 bandpass filter, the overall average value of $M_{r\text{\_}c}$ for 100% cotton is 0.69, with a maximum deviation of 2%. The overall average value of $M_{r\text{\_}c}$ for 100% polyester is 1.0, with a maximum deviation of 3.5%. The large separation in the mean value of the $M_{r\text{\_}c}$ for the fibre types means that the value of $M_{r\text{\_}c}$ can be used as a valid metric, distinguishable from noise, for identification of these fibre materials. Based on the value of this metric, an easy and direct distinction between selected fibre types can be determined. This can be achieved by defining the value ranges for each of the materials.

#### 4.2.3. Effect of Carbon Black Dye on the NIR Images

**Figure 14:**
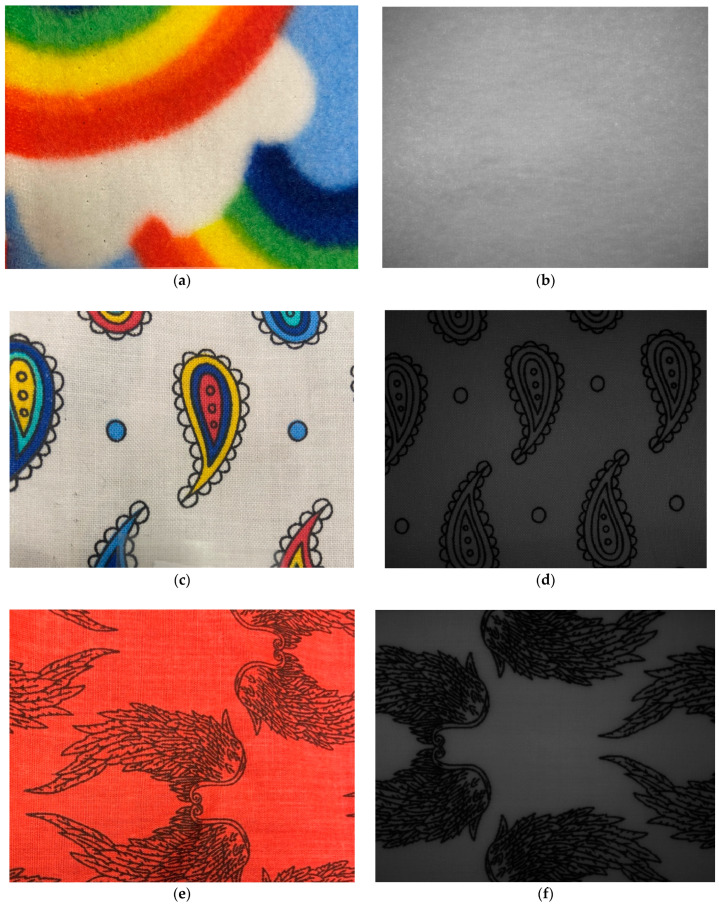
Photos and corresponding images from an NIR camera with Bi1550 bandpass filter attached: (**a**) AA20 photo; (**b**) AA20 NIR image; (**c**) AA39 photo; (**d**) AA39 NIR image; (**e**) AA33 photo; (**f**) NIR image.

Figure 14 shows three new samples of cotton and polyester materials. Figure 14a shows the photo of the textile sample with rainbow colours. Figure 14b, shows the corresponding sample’s image captured by the NIR camera when the Bi1550 bandpass filter was attached to the camera lens. This filter removes any colour information reaching the NIR camera sensor.

Graphite-based dyes absorb most of the light in the sensitive range of the NIR camera, which is between 400 nm to 1700 nm. As seen from Figure 14, the colour information is not transmitted to the camera’s sensor, as the graphite-based dye absorbs most of the light in the sensitive range of the camera. The patterns where graphite is present in the dye appear as black and are easily detectable.

The NIR camera can be used to detect graphite-based dyes in textiles due to the resulting “masking effect” of the dye on the material underneath the dye.

The carbon black dye acts as a layer obstructing the material characteristics data to be detected by the imaging technologies we are using. Effectively, the system cannot detect the type of material underneath the areas covered by the carbon black dye. However, if the entire material is not completely covered by the carbon black dye, there will exist regions which can be used for material identification. Using simple image-processing techniques, any areas covered by the dye can be ignored, and the material identification process can be carried out in the remaining regions

**Figure 15:**
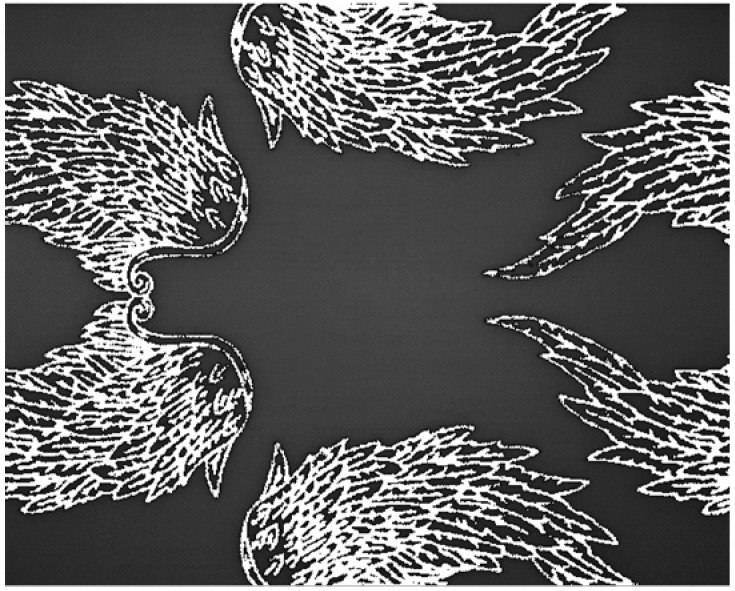
Removing the carbon black dye regions.

As discussed earlier, the proposed system in this paper ignores any overexposed pixels, with a value of 255, and any underexposed pixels with a value of 0. Therefore, any pixels in the areas covered by carbon black dye must be set to either an overexposed or underexposed value. Figure 15 shows the processed image from Figure 14f, with the carbon black dye covered regions set to the overexposed value of 255.

## 5. Conclusions

Hyperspectral imaging systems have been widely used for identification of materials in various recycling applications, including textiles. The system provides a large amount of data at each camera pixel according to the light reflectance which is an attribute of the material under inspection. Using these extensive data, models can be trained to identify the materials. The NIR camera, however, only provides limited data at each pixel, in the form of light intensity. This makes the output from the NIR cameras highly dependent on the camera exposure time and the physical spread of the light illumination across the image. It may be possible to process the images from the NIR camera to distinguish between two materials; however, this system would only function correctly as long as the parameters affecting the perceived illumination intensity remain unchanged. This also implies that the parameters used to identify materials are not transportable and have to be calculated for each specific illumination spread and camera exposure time setting. In this paper, a robust and repeatable methodology was proposed to overcome the shortcomings associated with the use of NIR cameras for materials identification.

A methodology for the correction of uneven illumination of images captured via an NIR camera was proposed and investigated. The process involves several steps, including image processing as well as the use of statistical methodologies. Using the corrected images, several statistical measures were calculated and investigated to find a metric which could be used as an indicator for textile materials identification, independent of illumination across the image and the camera’s exposure time. A repeatable metric was identified and was tested using four textile samples. It was observed that the value of the $M_{r\text{\_}c}$, the ratio of the mean value of the corrected image to the mean value of the reference image, remained constant with a very small deviation, even under different illumination spreads and camera exposure times, thus providing a transportable and reliable metric which could be used for identification and distinction between textile materials.

It was also shown that the NIR camera could be used to detect dyes which are graphite-based. This would allow the separation of the regions with graphite-based dyes from other regions, using simple image-processing steps. Those regions without graphite-based dye present could then be used for the identification of the textile materials.

This work was focused on the identification of the constituent fibres in single material samples and did not investigate multi-materials blended samples. In future works, blended samples will be considered.

## Figures and Tables

**Table 1:** Average values of $M_{r\text{\_}c}$ and the maximum deviations for the BP1550 and Bi1550 filters for 100% cotton and 100% polyester.

Bandpass Filter	BP1550		Bi1550	
	Average Value of $M_{r\text{\_}c}$	Maximum Deviation	Average Value of $M_{r\text{\_}c}$	Maximum Deviation
100% Cotton	0.68	2%	0.65	4%
100% Polyester	1	1%	0.98	8%

## Data Availability

The data are contained within this article.

## References

[1] Mehrubeoglu M., Van Sickle A., Turner J. Detection and identification of plastics using SWIR hyperspectral imaging. Proceedings of the Imaging Spectrometry XXIV: Applications, Sensors, and Processing.

[2] Singh M.K., Hait S., Thakur A. (2023). Hyperspectral imaging-based classification of post-consumer thermoplastics for plastics recycling using artificial neural network. Process Saf. Environ. Prot..

[3] Moroni M., Mei A. (2020). Characterization and separation of traditional and bio-plastics by hyperspectral devices. Appl. Sci..

[4] Tamin O., Moung E.G., Dargham J.A., Yahya F., Omatu S. (2023). A review of hyperspectral imaging-based plastic waste detection state-of-the-arts. Int. J. Electr. Comput. Eng. (IJECE).

[5] Clothing Labels: Accurate or Not?. https://www.circle-economy.com/resources/clothing-labels-accurate-or-not.

[6] Liu Y., Tao F., Yao H., Kincaid R. (2023). Feasibility study of assessing cotton fiber maturity from near infrared hyperspectral imaging technique. J. Cotton Res..

[7] Cura K., Rintala N., Kamppuri T., Saarimäki E., Heikkilä P. (2022). Textile recognition and sorting for recycling at an automated line using near infrared spectroscopy. Recycling.

[8] Riba J.-R., Cantero R., Riba-Mosoll P., Puig R. (2022). Post-consumer textile waste classification through near-infrared spectroscopy, using an advanced deep learning approach. Polymers.

[9] Bonifazi G., Gasbarrone R., Palmieri R., Serranti S. (2024). A Characterization Approach for End-of-Life Textile Recovery Based on Short-Wave Infrared Spectroscopy. Waste Biomass Valorization.

[10] What Is NIR and SWIR Spectroscopy?. https://andor.oxinst.com/learning/view/article/what-is-nir-and-swir-spectroscopy.

[11] NIR and SWIR Questions & Answers. https://hub.hamamatsu.com/us/en/ask-an-engineer/imaging/nir-and-swir-questions-and-answers.html.

[12] Sun X., Zhou M., Sun Y. (2016). Classification of textile fabrics by use of spectroscopy-based pattern recognition methods. Spectrosc. Lett..

[13] Riba J.-R., Puig R., Cantero R. (2023). Portable Instruments Based on NIR Sensors and Multivariate Statistical Methods for a Semiautomatic Quality Control of Textiles. Machines.

[14] Tripathi S.K., Gupta B., Tiwari M. (2020). An alternative approach to preserve naturalness with non-uniform illumination estimation for images enhancement using normalized L 2-Norm based on Retinex. Multidimens. Syst. Signal Process..

[15] Lu Y., Tanaka M., Kawakami R., Okutomi M. (2023). Local Brightness Normalization for Image Classification and Object Detection Robust to Illumination Changes. Pacific-Rim Symposium on Image and Video Technology.

[16] Lv F., Liu B., Lu F. Fast enhancement for non-uniform illumination images using light-weight CNNs. Proceedings of the 28th ACM International Conference on Multimedia.

[17] Basler Ace 2 X a2A1280-80gmSWIR. https://www.baslerweb.com/en/shop/a2a1280-80gmswir/.

[18] Computar: M1218-APVSW. https://www.computar.com/products/m1218-apvsw.

[19] Midwest Optical Systems Inc. Bi1300 Short-Wave Infrared Bandpass Filter. https://midopt.com/filters/bi1300/.

[20] Midwest Optical Systems Inc. Bi1550 Short-Wave Infrared Bandpass Filter. https://midopt.com/filters/bi1550/.

[21] Midwest Optical Systems Inc. BP1550 Short-Wave Infrared Bandpass Filter. https://midopt.com/filters/bp1550/.

